# Arylamine *N*-acetyltransferase polymorphisms in Han Chinese patients with ankylosing spondylitis and their correlation to the adverse drug reactions to sulfasalazine

**DOI:** 10.1186/2050-6511-15-64

**Published:** 2014-11-21

**Authors:** Zhi-duo Hou, Zheng-yu Xiao, Yao Gong, Yu-ping Zhang, Qing Yu Zeng

**Affiliations:** Department of Rheumatology, the First Affiliated Hospital of Shantou University Medical College, No.57 Chang Ping Road, Shantou, 515041 Guangdong Province China; Research Unit of Rheumatology, Shantou University Medical College, No.22 Xin Ling Road, Shantou, 515041 Guangdong Province China

**Keywords:** Arylamine *N*-acetyltransferases, Genetic polymorphism, Sulfasalazine, Ankylosing spondylitis, Adverse drug reactions

## Abstract

**Background:**

Polymorphisms of Arylamine *N-*acetyltransferase (*NAT*) that contribute to diverse susceptibilities of some autoimmune diseases are also linked to the metabolism of several drugs including sulfasalazine (SSZ). The aim of this study was to investigate the distribution of *NAT* polymorphisms in Han Chinese patients with ankylosing spondylitis (AS) and their correlation to sulfasalazine-induced adverse drug reactions (ADRs).

**Methods:**

Arylamine *N*-acetyltransferase 1 (*NAT1*) and arylamine *N*-acetyltransferase 2 (*NAT2*) genotypes were determined in 266 AS patients who received SSZ treatment and 280 healthy controls. The correlation between *NAT* polymorphisms and SSZ-induced ADRs was analyzed.

**Results:**

The co-occurrence frequency of *NAT2* fast acetylator genotype and NAT1*10/NAT1*10 genotype was lower in AS patients than in controls. No positive correlations were detected between *NAT* polymorphisms and AS clinical features. The prevalence of SSZ-induced ADRs and drug withdrawal was 9.4% and 7.1%, respectively. The frequencies of overall ADRs, dose-related ADRs, and termination of drug treatment because of intolerance were higher in the *NAT2* slow acetylator genotype carriers than in the fast-type carriers and in those with co-existence of *NAT1* and *NAT2* slow acetylator genotypes. Furthermore, the ADRs emerged earlier in the AS cases carrying both *NAT1* and *NAT2* slow acetylator genotypes.

**Conclusions:**

The prevalence of co-occurring *NAT2* fast acetylator genotype and NAT1*10/NAT1*10 genotype was lower in AS patients than in controls. The *NAT2* slow acetylator genotype and co-existing *NAT1* and *NAT2* slow acetylator genotypes appear to be associated with higher risks of SSZ-induced ADRs.

## Background

Sulfasalazine (SSZ) is one of the disease-modifying antirheumatic drugs (DMARDs) and has been widely used for the treatment of ankylosing spondylitis (AS) in China for years [1]. Though the efficacy of SSZ in treating AS is still under debate, Chinese rheumatologists prefer to prescribe SSZ for the treatment of AS because of its good cost-effectiveness ratio. Nevertheless, the therapy is often terminated in about 14–38% of AS patients receiving SSZ treatment because of adverse drug reactions (ADRs) [2–4]. There is currently no effective method of identifying a patient’s susceptibility to SSZ-induced ADRs. Thus, predicting the therapeutic response of AS patients to SSZ is of great importance for individualized therapies of AS.

SSZ is constituted by 5-aminosalicylic acid (5-ASA) and sulfapyridine (SP). SP is considered to be an active component for the therapy of AS [5], and is related to ADRs such as nausea, vomiting, and headache [6], while 5-ASA is effective for the treatment of inflammatory bowel disease and is related to ADRs such as diarrhea [7]. When orally administered, SSZ is metabolized into SP and 5-ASA in the presence of the gut microbiota [8, 9]. SP is almost completely absorbed and metabolized to *N*-acetyl-SP predominantly by hepatic arylamine *N*-acetyltransferase 2 (NAT2), while only about 20–30% of 5-ASA is absorbed and metabolized to *N*-acetyl-5-ASA by arylamine *N*-acetyltransferase 1 (NAT1), the rest being eliminated in the feces [9, 10]. Therefore, arylamine *N*-acetyltransferases (NATs) play an important role in the metabolism of SP and 5-ASA.

Humans have two functional *NAT* genes (*NAT1* and *NAT2*) and a pseudogene (*NATP*), all found on chromosome 8p22 [11]. Genetic mutations of *NAT1* and *NAT2* result in amino acid substitutions of their protein products, and may lead to altered gene expression and activity of the NAT enzyme [11–14]. These changes were shown to contribute to increased susceptibility to a range of disorders including autoimmune diseases [15–20] and carcinomas [21–24], but were also linked to the differences in the ability to *N*-acetylate certain drugs including SSZ [6, 10, 25–29]. In this study, we investigated the genotypes of the two human *NATs* in Han Chinese patients with AS *versus* healthy controls and also the potential correlation between *NAT* polymorphisms and SSZ-induced ADRs.

## Methods

### Subjects

Two hundred and sixty-six consecutive cases of AS from the First Affiliated Hospital of Shantou University Medical College were studied during the period of 2004–2010. All patients were diagnosed based on the Modified New York Criteria of AS [30]. All patients were treated with SSZ at a dose of 1.5–3.0 g/day (Shanghai Zhongxi Sunve Pharmaceutical Co., Ltd, China). Non-steroidal anti-inflammatory drugs were prescribed as needed. Two hundred and eighty healthy volunteers served as the healthy controls. All subjects were Han Chinese residing in Shantou, China. This study was approved by the Institutional Ethics Committee of Shantou University Medical College, and written informed consent was obtained from all participants.

### Determination of *NAT*genetic polymorphisms

Genomic DNA was extracted from peripheral blood samples with the QIAamp Blood Kit (QIAGEN, Hilden, Germany) and stored at -80°C. Genetic polymorphisms of *NAT1* (NAT1*4, NAT1*3, NAT1*10, NAT1*11) and *NAT2* (NAT2*4, NAT2*5, NAT2*6, NAT2*7) were determined using the polymerase chain reaction-restriction fragment length polymorphism (PCR-RFLP) method [24, 31]. The *NAT* genotypes of a number of samples were confirmed through direct DNA sequencing for quality control purposes. Subjects were classified as *NAT1* fast acetylator genotype carriers (those with at least one NAT1*10 allele) or *NAT1* slow acetylator genotype carriers (those without NAT1*10 allele) [24]. Individuals carrying at least one wild-type allele (NAT2*4 allele) were classified as *NAT2* fast acetylator genotype carrier, and those carrying the mutant allele (NAT2*5, *6, *7) were classified as *NAT2* slow acetylator genotype carriers [32].

### Observation of clinical features

Clinical features observed included age of onset, duration of disease, joint involvement (classified into two types: axial joint involvement only, and both axial and peripheral joint involvements), incidence of radiological hip involvement, and HLA-B27 status.

### Assessment of ADRs

All patients were followed up for more than 2 years and SSZ-induced ADRs were monitored. ADRs and severe ADRs were defined according to the criteria proposed by Edwards et al. [33]. ADRs were classified into six types: dose-related (Augmented), non-dose-related (Bizarre), dose-related and time-related (Chronic), time-related (Delayed), withdrawal (End of use), and failure of therapy (Failure). The causality assessment of suspected ADRs was determined as described previously [33]. ADRs were classified as being “certain”, “probable”, “possible”, “unlikely”, “conditional”, and “unassessable”. All ADRs noted in this study were “certain”, “probable”, or “possible”.

### Statistical analysis

Data were analyzed using SPSS 19.0 statistical software. Departure from the Hardy–Weinberg equilibrium was tested by the chi-squared test. Frequencies of the alleles, genotypes, acetylator genotypes, and co-occurrence of *NAT1* with *NAT2* acetylator genotypes in each group were compared using chi-squared test or the Fisher’s exact test as appropriate. Age of onset and duration of disease in each group were compared using t-test, one-way ANOVA test or Kruskal Wallis test as appropriate. Odds ratio (OR) and a 95% confidence interval (95% CI) were estimated from logistic regression models to test for associations between the risk of SSZ-induced ADRs and the *NAT* acetylator genotypes. Binary logistic regression analysis was used to identify the variables (*NAT* acetylator genotype, dose, age, sex, and NSAIDs combination) that provided an important contribution towards the variability of SSZ-induced ADRs and to adjust for confounding variables by analysis of covariates. The prevalence of drug treatment termination because of SSZ-induced ADRs in each group was compared using the chi-squared test or the Fisher’s exact test as appropriate. Differences in the duration of ADRs occurring in each group were compared using the Mann–Whitney U test. *P* < 0.05 was considered statistically significant.

## Results

### Subject characteristics

Of the AS patients, 219 were male and 47 were female. The positive rate of HLA-B27 in patients was 90.2%. The mean age was 27.8 ± 9.1 years and the mean disease duration was 6.8 ± 5.7 years. The control group included 221 male and 59 female healthy volunteers with a mean age of 35.1 ± 10.0 years.

### *NAT*polymorphisms in AS patients and healthy controls

The distribution of *NAT1* and *NAT2* gene polymorphisms in AS patients and healthy controls is shown in Table 1. The allele frequencies of both AS patients and healthy controls were consistent with the Hardy–Weinberg equilibrium (*P* > 0.05). No significant differences between the AS and control group were found for the distribution of alleles, genotypes, and acetylator genotypes when *NAT1* and *NAT2* polymorphisms were analyzed independently. However, the co-occurrence frequency of the *NAT2* fast acetylator genotype and NAT1*10/NAT1*10 genotype was lower in AS patients than in controls (6.4% *vs* 11.4%, *P* = 0.04, OR = 0.53, 95% CI 0.29–0.98).

**Table 1:** **Distribution of** ***NAT*** **polymorphisms in AS patients and healthy controls**

Alleles/Genotypes/Acetylator genotypes		AS		HC		χ ^2^	***P***
		N	%	N	%		
*NAT1* Alleles	NAT1*4	268	50.4	261	46.6	1.55	0.21
	NAT1*3	101	19.0	108	19.3	0.02	0.90
	NAT1*10	159	29.9	188	33.6	1.71	0.19
	NAT1*11	4	0.7	3	0.5	-	0.72*
	Total	532		560			
*NAT1* Genotypes	NAT1*4/NAT1*4	75	28.2	65	23.2	1.78	0.18
	NAT1*4/NAT1*3	39	14.7	48	17.1	0.63	0.43
	NAT1*4/NAT1*10	77	28.9	80	28.6	0.01	0.92
	NAT1*4/NAT1*11	2	0.8	3	1.1	-	1.00*
	NAT1*3/NAT1*3	14	5.2	14	5.0	0.02	0.89
	NAT1*3/NAT1*10	34	12.8	32	11.4	0.24	0.63
	NAT1*3/ NAT1*11	0	0	0	0	-	-
	NAT1*10/NAT1*10	23	8.6	38	13.6	3.33	0.07
	NAT1*10/NAT1*11	2	0.8	0	0	-	-
	NAT1*11/NAT1*11	0	0	0	0	-	-
	Total	266		280			
*NAT1* Acetylator Genotypes	Fast	136	51.1	150	53.6	0.33	0.57
	Slow	130	48.9	130	46.4		
	Total	266		280			
*NAT2* Alleles	NAT2*4	310	58.3	340	60.7	0.68	0.41
	NAT2*5	18	3.4	18	3.2	0.02	0.88
	NAT2*6	134	25.2	129	23.0	0.69	0.41
	NAT2*7	70	13.1	73	13.1	0.00	0.95
	Total	532		560			
*NAT2* Genotypes	NAT2*4/NAT2*4	98	36.8	110	39.3	0.35	0.56
	NAT2*4/NAT2*5	9	3.4	10	3.6	0.01	0.91
	NAT2*4/NAT2*6	70	26.3	71	25.4	0.07	0.80
	NAT2*4/NAT2*7	35	13.2	39	13.9	0.07	0.79
	NAT2*5/NAT2*5	0	0	1	0.4	-	-
	NAT2*5/NAT2*6	6	2.3	2	0.7	-	0.17*
	NAT2*5/NAT2*7	3	1.1	4	1.4	-	1.00*
	NAT2*6/NAT2*6	18	6.8	18	6.4	0.03	0.87
	NAT2*6/NAT2*7	22	8.2	20	7.1	0.24	0.62
	NAT2*7/NAT2*7	5	1.9	5	1.8	-	1.00*
	Total	266		280			
*NAT2* Acetylator Genotypes	Fast	212	79.7	230	82.1	0.53	0.47
	Slow	54	20.3	50	17.9		
	Total	266		280			
*NAT2* fast acetylator genotype plus NAT1*10/NAT1*10		17	6.4	32	11.4	4.24	0.04

*Results of Fisher’s exact test; AS = Ankylosing Spondylitis; HC = healthy control; *NAT1* = Arylamine *N*-acetyltransferases 1; *NAT2* = Arylamine *N*-acetyltransferases 2; N = Number; χ^2^ = Chi-squared test.

### Correlations between *NAT*polymorphisms and clinical features of AS

The age of onset, duration of disease, joint involvement types, incidence of radiological hip involvement, and the positive rate of HLA-B27 were not significantly different in each *NAT* genotype or *NAT* acetylator genotype group among the AS patients (*P* > 0.05).

### SSZ-induced ADRs

SSZ-induced ADRs occurred in 25 cases (9.4%) of AS (Table 2). Among these, 16 patients (64.0%) experienced dose-related ADRs; eight patients (32.0%) experienced non-dose-related ADRs, and one patient (4.0%) experienced time-related ADR. The dose-related ADRs observed in this study included nausea, anorexia, abdominal pain, diarrhea, epigastric discomfort, dizziness, elevation of serum liver enzyme levels, and chest congestion. Non-dose-related ADRs included rash, oral ulcer, and leukopenia. One patient with cacospermia was classified as having time-related ADR. No serious ADRs were found in the AS group of patients. SSZ therapy was terminated because of SSZ-induced ADRs in 19 AS patients (7.1%). Of these, 10 patients (52.6%) withdrew from SSZ therapy because of dose-related ADRs; eight patients (42.1%) because of non-dose-related ADRs, and one patient (5.3%) because of time-related ADR. The mean duration of SSZ therapy from the time when the first ADR occurred was 19 days (range 3–450 days). Most ADRs occurred within 12 weeks after the start of SSZ treatment (23 cases, 92.0%).

**Table 2:** **Clinical features and** ***NAT*** **polymorphisms in 25 AS patients who experienced SSZ-induced ADRs**

ADR types*	Cases	SSZ Dose (g/day)	ADR	Termination of Drug therapy	***NAT2***		***NAT1***	
					Genotypes	Acetylator genotypes	Genotypes	Acetylator genotypes
**Dose-related**	Case 1	3.0	Nausea	No	NAT2*4/NAT2*7	Fast	NAT1*10/NAT1*10	Fast
	Case 2	3.0	Nausea, anorexia	No	NAT2*4/NAT2*5	Fast	NAT1*3/NAT1*10	Fast
	Case 3	1.5	Abdominal pain	Yes	NAT2*4/NAT2*6	Fast	NAT1*4/NAT1*10	Fast
	Case 4	3.0	Diarrhea	Yes	NAT2*7/NAT2*7	Slow	NAT1*4/NAT1*4	Slow
	Case 5	3.0	Diarrhea	No	NAT2*5/NAT2*7	Slow	NAT1*4/NAT1*4	Slow
	Case 6	3.0	Epigastric discomfort	No	NAT2*4/NAT2*4	Fast	NAT1*4/NAT1*10	Fast
	Case 7	1.5	Epigastric discomfort	Yes	NAT2*4/NAT2*7	Fast	NAT1*4/NAT1*10	Fast
	Case 8	1.5	Increase in transaminases	Yes	NAT2*6/NAT2*7	Slow	NAT1*4/NAT1*4	Slow
	Case 9#	2.25	Increase in transaminases	Yes	NAT2*6/NAT2*7	Slow	NAT1*4/NAT1*3	Slow
	Case 10	2.25	Dizziness	No	NAT2*6/NAT2*7	Slow	NAT1*3/NAT1*3	Slow
	Case 11	1.5	Dizziness	Yes	NAT2*6/NAT2*6	Slow	NAT1*4/NAT1*4	Slow
	Case 12	1.5	Dizziness	Yes	NAT2*5/NAT2*6	Slow	NAT1*4/NAT1*4	Slow
	Case 13	1.5	Dizziness	Yes	NAT2*4/NAT2*7	Fast	NAT1*10/NAT1*10	Fast
	Case 14	1.5	Dizziness	Yes	NAT2*6/NAT2*6	Slow	NAT1*4/NAT1*4	Slow
	Case 15	3.0	Dizziness	No	NAT2*4/NAT2*4	Fast	NAT1*4/NAT1*4	Slow
	Case 16	1.5	Chest congestion	Yes	NAT2*5/NAT2*6	Slow	NAT1*4/NAT1*3	Slow
**Non-dose-related**	Case 17	2.25	Leukopenia	Yes	NAT2*4/NAT2*6	Fast	NAT1*4/NAT1*4	Slow
	Case 18	1.5	Rash	Yes	NAT2*6/NAT2*6	Slow	NAT1*4/NAT1*4	Slow
	Case 19	1.5	Rash	Yes	NAT2*4/NAT2*6	Fast	NAT1*3/NAT1*10	Fast
	Case 20	1.5	Rash	Yes	NAT2*4/NAT2*6	Fast	NAT1*4/NAT1*11	Slow
	Case 21	3.0	Rash	Yes	NAT2*4/NAT2*4	Fast	NAT1*4/NAT1*10	Fast
	Case 22	3.0	Rash	Yes	NAT2*4/NAT2*4	Fast	NAT1*3/NAT1*10	Fast
	Case 23	2.25	Oral ulcer	Yes	NAT2*4/NAT2*4	Fast	NAT1*10/NAT1*10	Fast
	Case 24	2.25	Oral ulcer	Yes	NAT2*4/NAT2*4	Fast	NAT1*4/NAT1*10	Fast
**Time-related**	Case 25#	2.25	Cacospermia	Yes	NAT2*4/NAT2*6	Fast	NAT1*3/NAT1*3	Slow

*ADRs were classified into six types: dose-related, non-dose-related, dose-related and time-related, time-related, withdrawal (End of use), and failure of therapy; #Case 9 was observed to have elevated serum liver enzyme levels at day 138 and case 25 had cacospermia at day 450 after the start of SSZ treatment; ADRs = Adverse Drug Reactions; *NAT1* = Arylamine *N*-acetyltransferases 1; *NAT2* = Arylamine *N*-acetyltransferases 2; SSZ = Sulfasalazine.

### Correlations of *NAT*polymorphisms and SSZ-induced ADRs

Correlations between *NAT* acetylator genotypes and SSZ-induced ADRs are shown in Table 3. The incidence of overall ADRs in patients carrying the *NAT2* slow acetylator genotype was 18.5% (10/54) and was significantly higher than patients carrying the *NAT2* fast acetylator genotype (7.1%, 15/212, *P* = 0.013, OR = 2.99, 95% CI 1.26-–7.09). The incidence of dose-related ADRs in patients carrying the *NAT2* slow acetylator genotype was also higher than in patients carrying the fast acetylator genotype (16.7% *vs* 3.3%, *P* = 0.002, OR = 5.17, 95% CI 1.79-–14.92). No significant difference in the incidence of non-dose-related ADRs was found between the *NAT2* fast and *NAT2* slow acetylator genotype groups (3.3% *vs* 1.9%, *P* > 0.05). The prevalence of drug treatment termination owing to ADRs or dose-related ADRs was higher in the *NAT2* slow acetylator genotype carriers than in the fast-type carriers (14.8% *vs* 5.2%, *P* = 0.032 and 13.0% *vs* 1.4%, *P* = 0.001, respectively).

**Table 3:** **Associations between** ***NAT*** **acetylator genotypes and SSZ-induced ADRs**

	***NAT2***acetylator genotype		***NAT1***acetylator genotype		Co-existence of ***NAT1***and ***NAT2***acetylator genotypes	
	Fast	Slow	Fast	Slow	***NAT1***slow plus ***NAT2***slow	Non ***NAT1***slow plus ***NAT2***slow
Total ADRs	7.1%(15/212)	18.5%(10/54)	8.1%(11/136)	10.8%(14/130)	26.3%(10/38)	6.6%(15/228)
P*	0.013		0.45		<0.001	
OR*	2.99				5.07	
95% CI*	1.26-7.09				2.08-12.37	
Drug therapy termination rate due to ADRs	5.2%(11/212)	14.8%(8/54)	5.9%(8/136)	8.5%(11/130)	21.1%(8/38)	4.8%(11/228)
*P*	0.032#		0.41		0.002#	
Dose-related ADR	3.3%(7/212)	16.7%(9/54)	4.4%(6/136)	7.7%(10/130)	23.7%(9/38)	3.1%(7/228)
P*	0.002		0.27		<0.001	
OR*	5.17				9.52	
95% CI*	1.79-14.92				3.20-28.30	
Drug therapy termination rate due to dose-related ADRs	1.4%(3/212)	13.0%(7/54)	2.2%(3/136)	5.4%(7/130)	18.4%(7/38)	1.3%(3/228)
*P*	0.001#		0.21#		<0.001#	
Non-dose-related ADR	3.3%(7/212)	1.8%(1/54)	3.7%(5/136)	2.3%(3/130)	2.6%(1/38)	3.1%(7/228)
P*	0.58		0.51		0.88	

*Results of binary logistic regression analysis after adjusting for dose, age, sex, and NSAIDs combination; #Results of Fisher’s exact test; OR = odds ratio; CI = confidence interval; ADRs = Adverse Drug Reactions; *NAT1* = Arylamine *N*-acetyltransferases 1; *NAT2* = Arylamine *N*-acetyltransferases 2; SSZ = Sulfasalazine.

The incidences of overall ADRs, dose-related ADRs, and non-dose-related ADRs in patients carrying the *NAT1* fast acetylator genotype were 8.1% (11/136), 4.4% (6/136), and 3.7% (5/136) respectively, similar to 10.8% (14/130), 7.7% (10/130), and 2.3% (3/130) in patients carrying the *NAT1* slow acetylator genotype (*P* > 0.05). The prevalence of drug treatment termination owing to overall ADRs or dose-related ADRs was slightly higher in the *NAT1* slow acetylator genotype carriers than in the fast-type carriers (8.5% *vs* 5.9%, and 5.4% *vs* 2.2%, respectively). However, the difference was not significant (*P* > 0.05).

The incidences of both overall ADRs and dose-related ADRs were higher in patients carrying both the *NAT1* slow acetylator genotype and the *NAT2* slow acetylator genotype than in the other carriers (26.3% *vs* 6.6%, *P* < 0.001, OR = 5.07, 95% CI 2.08–12.37 and 23.7% *vs* 3.1%, *P* < 0.001, OR = 9.52, 95% CI 3.20–28.30). The prevalence of drug treatment termination owing to overall ADRs or dose-related ADRs was also higher in the carriers of both *NAT1* slow acetylator genotype and *NAT2* slow acetylator genotype than in the other carriers (21.1% *vs* 4.8%, *P* = 0.002 and 18.4% *vs* 1.3%, *P* < 0.001, respectively). The mean duration of SSZ treatment when the first ADR occurred in patients carrying both the *NAT1* slow acetylator genotype and the *NAT2* slow acetylator genotypes was 8 days (range 3–128 days), shorter than that of 33 days (range 10–450 days) in the carriers of other acetylator genotypes (*P* = 0.003). No significant differences in the frequencies of non-dose-related ADRs were found among carriers of any *NAT1* and *NAT2* acetylator genotype combinations (*P* > 0.05).

Only one patient was identified that experienced time-related ADR and carried *NAT1* slow acetylator (NAT1*3/NAT1*3) and *NAT2* fast acetylator (NAT2*4/NAT2*6) genotypes.

## Discussion

This study showed that the *NAT* genotype appeared not to correlate with the development of AS or the clinical features of AS such as age of onset, duration of disease, joint involvements, incidence of radiological hip involvement, or positive status of HLA-B27. However, the frequency of co-occurrence of the *NAT2* fast acetylator genotype and the NAT1*10/NAT1*10 genotype was significantly lower in AS patients than in healthy controls (*P* = 0.04, OR = 0.53, 95% CI 0.29–0.98). This suggests that the co-occurrence of the *NAT2* fast acetylator genotype and the NAT1*10/NAT1*10 genotype is likely to reduce one-half the risk of AS in the Chinese Han population. It was reported that partial linkage disequilibrium exists between the NAT1*10 allele and the *NAT2* fast acetylation genotype, and a reduced risk for bladder cancer was identified in carriers of these co-existing genotypes [34]. Because the sample size of our study was relatively small, further investigation should be conducted to reveal the biological significance of the partial linkage disequilibrium between the NAT1*10 allele and the *NAT2* fast acetylation genotype in the development of AS.

In the current study, the most common SSZ-induced ADR were gastrointestinal reactions (nausea and diarrhea), followed by rash, dizziness, and elevated levels of serum liver transaminases. Hematological disturbances (leukopenia) and cacospermia were rarely seen. These characteristics were similar to those reported in previous studies [9, 35]. It was also reported that most SSZ-induced ADRs occurred within the first 12 weeks of SSZ treatment [36]. In our study, most ADRs (92%) also occurred within the first 12 weeks. However, one patient (case 9) was observed to have elevated serum liver enzyme levels at day 138 and another patient (case 25) had cacospermia at day 450 after the start of SSZ treatment. This indicates it is important to monitor the ADRs during the first 12 weeks of SSZ treatment. However, liver function tests need to be carried out beyond 12 weeks. Morphological examination and motility tests for sperm are also important for male patients.

The association between single nucleotide polymorphisms (SNPs) and *NAT* expression has been described in previous reports [11–14]. For example, 341 T > C (I114T) and 590G > A (R197Q) reduce NAT2 catalytic activities, whereas 481C > T (L161L) do not. *NAT* polymorphisms linked to the differences in the ability to *N*-acetylate SSZ have been identified [6, 10, 25–29]. However, it is still not clear whether the genetic variants of *NAT1* and *NAT2* underlie the patients’ response to SSZ. Previous studies identified a strong association between *NAT2* polymorphisms and SSZ-induced ADRs [20, 31, 37–39] and this association was thought to have an effect on the efficacy of SSZ [26, 39] . However, others suggested that *NAT1* and *NAT2* polymorphisms played no roles in predicting responses to SSZ or related toxicities [40, 41]. In our study, the frequencies of overall ADRs and dose-related ADRs of SSZ were higher in the *NAT2* slow acetylation genotype group than in the *NAT2* fast-type group. The ORs were 2.99 (95% CI 1.26–7.09) and 5.17 (95% CI 1.79–14.92), respectively, indicating that the *NAT2* slow acetylator genotype may be a risk factor for ADRs, especially the dose-related ADRs of SSZ. In addition, the prevalence of SSZ treatment terminations owing to overall ADRs or dose-related ADRs was also higher in the *NAT2* slow acetylator genotype carriers as compared with that in the *NAT2* fast-type carriers (14.8% *vs* 5.2%, *P* = 0.032 and 13.0% vs 1.4%, *P* = 0.001). In our study, non-dose-related ADRs only occurred in one patient carrying the *NAT2* slow acetylator genotype. Therefore, it is difficult to evaluate the contribution of the *NAT2* acetylation genotype to non-dose-related ADRs of SSZ.

The NAT1 function is also widely variable in human populations, although the effects of its genetic polymorphisms are not generally as marked as those of NAT2 [12, 13, 42]. The current study provided little evidence of correlation between *NAT1* acetylator genotypes and the risk of SSZ-induced ADRs by *NAT1* single-gene analysis. However, the co-existence of the *NAT1* slow acetylator genotype and the *NAT2* slow acetylator genotype conferred a 5-fold greater risk for overall ADRs, and a 9-fold greater risk for dose-related ADRs as compared with other acetylator genotype carriers. The termination of SSZ treatment owing to overall ADRs or dose-related ADRs was more commonly seen in AS patients carrying the *NAT1* slow and *NAT2* slow acetylator genotypes. Additionally, the onset of ADRs was earlier in patients with co-existing the *NAT1* slow and *NAT2* slow acetylator genotypes than that for other acetylator genotype carriers (8 *vs* 33 days, *P* = 0.003). These findings allow the identification of patients at a possible risk of SSZ-induced ADRs or subsequent terminations of SSZ treatment. It is also important to note that a multi-gene analysis approach is still worthwhile in pharmacogenetic studies even if no positive correlations are found *via* a single gene analysis.

Similar to the findings of other studies involving Asians such as Chinese and Japanese populations [19, 43], our results showed the prevalence of the *NAT2* slow acetylator genotype was 20.3% in Han Chinese patients with AS, which is significantly lower than that reported for Western populations (40–70%) [13]. This might explain why SSZ-induced ADRs are much less prevalent among the Chinese (9.4% in our study population) and the Japanese (11.1%) [37] as compared with Western populations (22.7-53.5%) [2, 9, 35, 40]. However, the statistical power is relatively low because of the low frequency of the *NAT2* slow acetylator genotype in our study population. It is a challenge to evaluate correlations between *NAT* polymorphisms and SSZ-induced ADRs without bias. Thus, studies of multi-ethnicity and larger populations are required.

## Conclusions

The co-occurrence of the *NAT2* fast acetylator genotype and the NAT1*10/NAT1*10 genotype is less frequent in AS patients compared with healthy controls in the Han Chinese population*.* The *NAT2* slow acetylator genotype and co-existing *NAT1* and *NAT2* slow acetylator genotypes appear to be associated with higher risks of SSZ-induced ADRs.

## Abbreviations

NAT: Arylamine *N*-acetyltransferases
NAT1: Arylamine *N*-acetyltransferases 1
NAT2: Arylamine *N*-acetyltransferases 2
AS: Ankylosing Spondylitis
ADRs: Adverse Drug Reactions
SSZ: Sulfasalazine
DMARDs: Disease-Modifying Antirheumatic Drugs
OR: Odds Ratio
CI: Confidence Interval.

## References

[1] Zeng QY (2003). Ankylosing spondylitis in Shantou, China: 15 years' clinical experience. J Rheumatol.

[2] Dougados M, Maetzel A, Mijiyawa M, Amor B (1992). Evaluation of sulphasalazine in the treatment of spondyloarthropathies. Ann Rheum Dis.

[3] Clegg DO, Reda DJ, Abdellatif M (1999). Comparison of sulfasalazine and placebo for the treatment of axial and peripheral articular manifestations of the seronegative spondylarthropathies: a Department of Veterans Affairs cooperative study. Arthritis Rheum.

[4] Schmidt WA, Wierth S, Milleck D, Droste U, Gromnica-Ihle E (2002). Sulfasalazine in ankylosing spondylitis: a prospective, randomized, double-blind placebo-controlled study and comparison with other controlled studies. Z Rheumatol.

[5] Taggart AGP, McEvoy FHR, Bird H (1996). Which is the active moiety of sulfasalazine in ankylosing spondylitis? A randomized, controlled study. Arthritis Rheum.

[6] Tanigawara Y, Kita T, Aoyama N, Gobara M, Komada F, Sakai T, Kasuga M, Hatanaka H, Sakaeda T, Okumura K (2002). N-acetyltransferase 2 genotype-related sulfapyridine acetylation and its adverse events. Biol Pharm Bull.

[7] Moum B (2008). Which are the 5-ASA compound side effects and how is it possible to avoid them. Inflamm Bowel Dis.

[8] Klotz U (1985). Clinical pharmacokinetics of sulphasalazine, its metabolites and other prodrugsof 5-aminosalicylic acid. Clin Pharmacokinet.

[9] Plosker GL, Croom KF (2005). Sulfasalazine: a review of its use in the management of rheumatoid arthritis. Drugs.

[10] Kuhn UD, Anschutz M, Schmucker K, Schug BS, Hippius M, Blume HH (2010). Phenotyping with sulfasalazine - time dependence and relation to NAT2 pharmacogenetics. Int J Clin Pharmacol Ther.

[11] Vatsis KP, Weber WW, Bell DA, Dupret JM, Evans DA, Grant DM, Hein DW, Lin HJ, Meyer UA, Relling MV, Sim E, Suzuki T, Yamazoe Y (1995). Nomenclature for N-acetyltransferases. Pharmacogenetics.

[12] Walker K, Ginsberg G, Hattis D, Johns DO, Guyton KZ, Sonawane B (2009). Genetic polymorphism in N-Acetyltransferase (NAT): Population distribution of NAT1 and NAT2 activity. J Toxicol Environ Health B Crit Rev.

[13] Meyer UA, Zanger UM (1997). Molecular mechanisms of genetic polymorphisms of drug metabolism. Annu Rev Pharmacol Toxicol.

[14] Hein DW (2009). N-acetyltransferase SNPs: emerging concepts serve as a paradigm for understandingcomplexities of personalized medicine. Expert Opin Drug Metab Toxicol.

[15] Pawlik A, Ostanek L, Brzosko I, Gawroska-Szklarz B, Brzosko M, Dabrowska-Zamojcin E (2002). Increased genotype frequency of N-acetyltransferase 2 slow acetylation inpatients with rheumatoid arthritis. Clin Pharmacol Ther.

[16] Baranska M, Trzcinski R, Dziki A, Rychlik-Sych M, Dudarewicz M, Skretkowicz J (2011). The role of N-acetyltransferase 2 polymorphism in the etiopathogenesis of inflammatory bowel disease. Dig Dis Sci.

[17] Mikuls TR, Levan T, Gould KA, Yu F, Thiele GM, Bynote KK, Conn D, Jonas BL, Callahan LF, Smith E, Brasington R, Moreland LW, Reynolds R, Gaffo A, Bridges SL (2012). Interactions of cigarette smoking with NAT2 polymorphisms on rheumatoid arthritis risk in African Americans. Arthritis Rheum.

[18] Tamer L, Tursen U, Eskandari G, Ates NA, Ercan B, Yildirim H, Atik U (2005). N-acetyltransferase 2 polymorphisms in patients with Behcet's disease. Clin Exp Dermatol.

[19] Kiyohara C, Washio M, Horiuchi T, Tada Y, Asami T, Ide S, Takahashi H, Kobashi G, Kyushu Sapporo SLE (KYSS) Study Group (2009). Cigarette smoking, N-acetyltransferase 2 polymorphisms and systemic lupuserythematosus in a Japanese population. Lupus.

[20] Gunnarsson I, Kanerud L, Pettersson E, Lundberg I, Lindblad S, Ringertz B (1997). Predisposing factors in sulphasalazine-induced systemic lupus erythematosus. Br J Rheumatol.

[21] García-Closas M, Malats N, Silverman D, Dosemeci M, Kogevinas M, Hein DW, Tardón A, Serra C, Carrato A, García-Closas R, Lloreta J, Castaño-Vinyals G, Yeager M, Welch R, Chanock S, Chatterjee N, Wacholder S, Samanic C, Torà M, Fernández F, Real FX, Rothman N (2005). NAT2 slow acetylation, GSTM1 null genotype, and risk of bladder cancer: results from the Spanish Bladder Cancer Study and meta-analyses. Lancet.

[22] Zhang J, Qiu LX, Wang ZH, Wang JL, He SS, Hu XC (2010). NAT2 polymorphisms combining with smoking associated with breast cancer susceptibility: a meta-analysis. Breast Cancer Res Treat.

[23] Butcher NJ, Minchin RF (2012). Arylamine N-acetyltransferase 1: a novel drug target in cancer development. Pharmacol Rev.

[24] Bell DA, Stephens EA, Castranio T, Umbach DM, Watson M, Deakin M, Elder J, Hendrickse C, Duncan H, Strange RC (1995). Polyadenylation polymorphism in the acetyltransferase 1 gene (NAT1) increases risk of colorectal cancer. Cancer Res.

[25] Kita T, Sakaeda T, Adachi S, Sakai T, Aoyama N, Hatanaka H, Kasuga M, Okumura K (2001). N-Acetyltransferase 2 genotype correlates with sulfasalazine pharmacokinetics after multiple dosing in healthy Japanese subjects. Biol Pharm Bull.

[26] Kumagai S, Komada F, Kita T, Morinobu A, Ozaki S, Ishida H, Sano H, Matsubara T, Okumura K (2004). N-Acetyltransferase 2 Genotype-Related Efficacy of Sulfasalazine in patients with Rheumatoid Arthritis. Pharm Res.

[27] Chen B, Zhang WX, Cai WM (2006). The influence of various genotypes on the metabolic activity of NAT2 in a Chinese population. Eur J Clin Pharmacol.

[28] Ma JJ, Liu CG, Li JH, Cao XM, Sun SL, Yao X (2009). Effects of NAT2 polymorphism on SASP pharmacokinetics in Chinese population. Clin Chim Acta.

[29] Daly AK (2013). Pharmacogenomics of adverse drug reactions. Genome Med.

[30] van der Linden S, Valkenburg HA, Cats A (1984). Evaluation of diagnostic criteria for ankylosing spondylitis. A proposal for modification of the New York criteria. Arthritis Rheum.

[31] Chen M, Xia B, Chen B, Guo Q, Li J, Ye M, Hu Z (2007). N-acetyltransferase 2 slow acetylator genotype associated with adverse effects of sulphasalazine in the treatment of inflammatory bowel disease. Can J Gastroenterol.

[32] Tanaka E, Taniguchi A, Urano W, Yamanaka H, Kamatani N (2004). Pharmacogenetics of disease-modifying anti-rheumatic drugs. Best Pract Res Clin Rheumatol.

[33] Edwards IR, Aronson JK (2000). Adverse drug reactions: definitions, diagnosis, and management. Lancet.

[34] Cascorbi I, Roots I, Brockmoller J (2001). Association of NAT1 and NAT2 polymorphisms to urinary bladder cancer: significantly reduced risk in subjects with NAT1*10. Cancer Res.

[35] Braun J, van der Horst-Bruinsma IE, Huang F, Burgos-Vargas R, Vlahos B, Koenig AS, Freundlich B (2011). Clinical efficacy and safety of etanercept versus sulfasalazine in patients with ankylosing spondylitis: a randomized, double-blind trial. Arthritis Rheum.

[36] Amos RS, Pullar T, Bax DE, Situnayake D, Capell HA, McConkey B (1986). Sulphasalazine for rheumatoid arthritis: toxicity in 774 patients monitored for one to 11 years. Br Med J (Clin Res Ed).

[37] Tanaka E, Taniguchi A, Urano W, Nakajima H, Matsuda Y, Kitamura Y, Saito M, Yamanaka H, Saito T, Kamatani N (2002). Adverse effects of sulfasalazine in patients with rheumatoid arthritis are associated with diplotype configuration at the N-acetyltransferase 2 gene. J Rheumatol.

[38] Soejima M, Kawaguchi Y, Hara M, Kamatani N (2008). Prospective study of the association between NAT2 gene haplotypes and severeadverse events with sulfasalazine therapy in patients with rheumatoid arthritis. J Rheumatol.

[39] Sabbagh N, Delaporte E, Marez D, Lo-Guidice JM, Piette F, Broly F (1997). NAT2 genotyping and efficacy of sulfasalazine in patients with chronic discoidlupus erythematosus. Pharmacogenetics.

[40] Ricart E, Taylor WR, Loftus EV, O'Kane D, Weinshilboum RM, Tremaine WJ, Harmsen WS, Zinsmeister AR, Sandborn WJ (2002). N-acetyltransferase 1 and 2 genotypes do not predict response or toxicity to treatment with mesalamine and sulfasalazine in patients with ulcerative colitis. Am J Gastroenterol.

[41] Kitas GD, Farr M, Waterhouse L, Bacon PA (1992). Influence of acetylator status on sulphasalazine efficacy and toxicity inpatients with rheumatoid arthritis. Scand J Rheumatol.

[42] Wang D, Para MF, Koletar SL, Sadee W (2011). Human N-acetyltransferase 1 *10 and *11 alleles increase protein expression through distinct mechanisms and associate with sulfamethoxazole-induced hypersensitivity. Pharmacogenet Genomics.

[43] Xie HG, Xu ZH, Ou-Yang DS, Shu Y, Yang DL, Wang JS, Yan XD, Huang SL, Wang W, Zhou HH (1997). Meta-analysis of phenotype and genotype of NAT2 deficiency in Chinese populations. Pharmacogenetics.

[CR44] The pre-publication history for this paper can be accessed here:http://www.biomedcentral.com/2050-6511/15/64/prepub

